# Control of Oxygen Vacancy Ordering in Brownmillerite Thin Films via Ionic Liquid Gating

**DOI:** 10.1021/acsnano.2c00012

**Published:** 2022-04-04

**Authors:** Hyeon Han, Arpit Sharma, Holger L. Meyerheim, Jiho Yoon, Hakan Deniz, Kun-Rok Jeon, Ankit K. Sharma, Katayoon Mohseni, Charles Guillemard, Manuel Valvidares, Pierluigi Gargiani, Stuart S. P. Parkin

**Affiliations:** † Max Planck Institute of Microstructure Physics, 06120 Halle (Saale), Germany; ‡ ALBA Synchrotron Light Source, E-08290 Cerdanyola del Vallès, Barcelona Spain

**Keywords:** oxygen vacancy channel, ionic liquid gating, strontium cobaltite, brownmillerite, strain

## Abstract

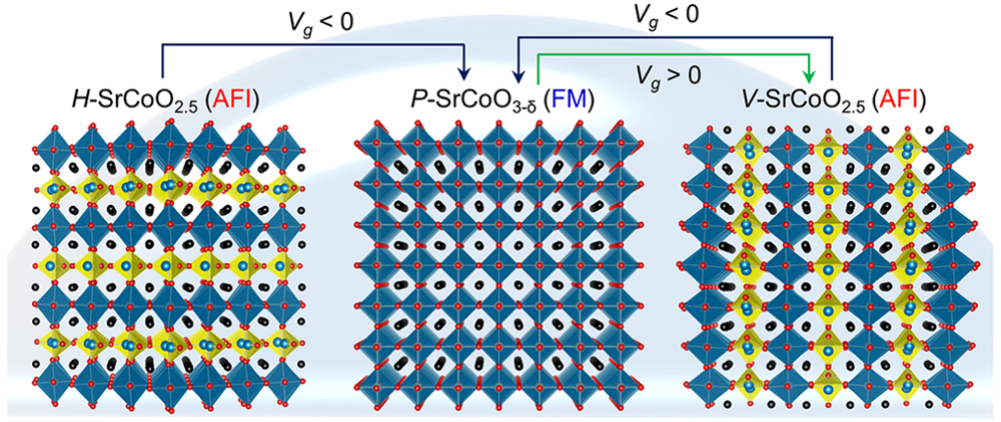

Oxygen defects and their atomic arrangements play a significant role in the physical properties of many transition metal oxides. The exemplary perovskite SrCoO_3-δ_ (*P-*SCO) is metallic and ferromagnetic. However, its daughter phase, the brownmillerite SrCoO_2.5_ (*BM-*SCO), is insulating and an antiferromagnet. Moreover, *BM-*SCO exhibits oxygen vacancy channels (OVCs) that in thin films can be oriented either horizontally (*H*-SCO) or vertically (*V*-SCO) to the film’s surface. To date, the orientation of these OVCs has been manipulated by control of the thin film deposition parameters or by using a substrate-induced strain. Here, we present a method to electrically control the OVC ordering in thin layers via ionic liquid gating (ILG). We show that *H*-SCO (antiferromagnetic insulator, AFI) can be converted to *P*-SCO (ferromagnetic metal, FM) and subsequently to *V*-SCO (AFI) by the insertion and subtraction of oxygen throughout thick films via ILG. Moreover, these processes are independent of substrate-induced strain which favors formation of *H*-SCO in the as-deposited film. The electric-field control of the OVC channels is a path toward the creation of oxitronic devices.

## Introduction

Oxygen vacancies play an important role in determining the physical properties of many transition-metal oxides (TMO). The oxygen vacancies can give rise to phase transitions, for example, between metallic and insulating states, and can significantly modify the electrical, magnetic, and optical properties.^1−8^ The oxygen vacancies also influence ionic transport that is key to the operation of devices for resistive switching and catalysis.^9−12^ In many cases, the oxygen vacancies form ordered arrays. An important example are the brownmillerite (*BM*) phases that are formed from the parent perovskite phase by the removal of oxygen in alternate layers: representative examples are SrCoO_2.5_,^5,12−18^ BaInO_2.5_,^19^ La_1–*x*_Sr_*x*_CoO_3-δ_,^20−26^ SrFeO_2.5_,^27−31^ SrFe_1–*x*_Co_*x*_O_2.5_,^31^ and CaFeO_2.5_.^33−35^ In these phases, oxygen vacancy channels (OVCs) are formed within the oxygen deficient layers. The OVCs can provide paths for fast ionic diffusion^11^ or storage of ions,^13,33^ leading to applications in fuel cells and rechargeable batteries. In addition, the OVCs affect the resistive switching response in memristors^9,10^ and strongly affect magnetic anisotropy.^7,8^

In thin films, the OVCs can be formed either parallel or perpendicular to the substrate surface and thereby strongly influence the properties of the films. The OVC orientation can be affected by applying strain in thin films by suitable choice of the substrate on which the film is deposited,^21,22,26,31^ by the deposition conditions of the film growth,^28−30^ or by using an appropriate capping layer.^20^ However, all these methods are passive and cannot be employed to control the OVCs in films after they have been formed. Here we show a means of electrically controlling the formation of the OVCs in thin layers of the exemplary oxide SrCoO_*x*_ using ionic liquid gating (ILG) which has been shown to modify the properties of thin films of several TMO by the electric field induced removal or insertion of oxygen into the film lattice.^1−5^ The phase transformation between brownmillerite and perovskite is enabled by applying an electric-field even at room temperature owing to the low oxygen vacancy formation energy and the high oxygen vacancy diffusivity of such systems.^24,25^

SrCoO_*x*_ is a fascinating material. Whereas the parent perovskite SrCoO_3-δ_ (*P*-SCO) is a ferromagnetic metal (FM), brownmillerite SrCoO_2.5_ (*BM*-SCO) is an antiferromagnetic insulator (AFI). It has previously been shown that ILG of SrCoO_*x*_ leads to a reversible transition between the *BM-*SCO and *P-*SCO phases.^5,16^ Here we demonstrate that *BM-*SrCoO_2.5_ with horizontal OVCs (*H-*SCO) can be transformed to the perovskite phase (*P-*SCO) that has no OVCs, and that *P-*SCO can be transformed to *BM-*SCO with vertical OVCs (*V-*SCO) by ILG. *V*-SCO can transform back to *P*-SCO but not to *H*-SCO. Notably, these transformations are shown to be independent of strain induced in SrCoO_*x*_ thin films by growing them on three distinct substrate materials. Our findings provide a method for the control of the OVCs in thin oxide films via ILG.

## Results and Discussion

Epitaxial, 30 nm thick, films of *BM-*SCO and *P-*SCO were grown on SrTiO_3_ (STO) (001) substrates using pulsed laser deposition (PLD). (La_0.18_Sr_0.82_)(Al_0.59_Ta_0.41_)O_3_ (LSAT) and LaAlO_3_ (LAO) (001) substrates were also used to investigate strain effects, as discussed later. The films were then gated by ILG at different gate voltages. Details are given in Supporting Information (SI) Figure S1 and the Methods section. SI Figure S2 shows X-ray diffraction (XRD) and resistivity measurements at room temperature for *BM-*SCO and *P-*SCO films after gating at a sequence of gate voltages for 1.5 h at each voltage. Bulk *BM*-SCO (related to *H*-type SCO as discussed below) crystallizes in an orthorhombic unit cell with the space group *Ima*2, characterized by lattice parameters, *a* = 5.470 Å, *b* = 5.574 Å, and *c* = 15.745 Å,^36^ while bulk *P*-SCO has a cubic perovskite unit cell with the space group *Pm*3̅*m*, and *a* = 3.836 Å.^37^ Before ILG, *BM-*SCO films grown on STO (001) show distinct peaks indexed by (002), (006), (0010), that are consistent with the body-centered lattice related to the alternate stacking of octahedral CoO_6_ and oxygen deficient tetrahedral CoO_4_ layers along the [001] axis. The unit cell of the *BM*-SCO film can be considered as an approximate  superstructure with respect to the STO (001) substrate lattice (see below and SI). The observation of the (00L) reflections in the specular θ-2θ scans indicates the presence of *H-*SCO. By applying negative gate voltages exceeding a magnitude of 1.5 V (U> |−1.5 V|), the *H-*SCO structure is transformed to *P-*SCO, which goes in parallel with a significant decrease in resistivity. On the other hand, we observe an increase of the resistivity if *P-*SCO is gated at positive gate voltages. After gating above 1 V, we observe reflections which are related to *V*-type *BM*-SCO (110) and (220) peaks, while the reflections related to *H*-SCO are not evident. The *BM*-SCO (004) and (110) peak positions are located at nearly the same 2θ-angle of approximately 23°. Similarly, the *P*-type (008) and the *V*-type (220) peak positions are close near 2θ ∼46°. This makes it difficult to immediately recognize any phase change. However, because *H*-SCO reflections along (00L) are not observed, the peaks at 2θ ∼23° and ∼46° must correspond to *V*-type *BM*-SCO (110) and (220), respectively, thus indicating the ILG induced formation of *V*-SCO. To confirm this transformation we carried out nonspecular azimuthal phi (φ) scans (SI Figure S3). Considering that the *V*-type *BM*-SCO film is (110) oriented, the film surface was tilted to a polar chi (χ) angle of approximately 56°, which corresponds to the angle between the (110) plane parallel to the film surface and the (116) plane. The set of {116} reflections is observed versus φ following an in-plane 4-fold symmetry (SI Figure S3a). Thus, these nonspecular scans confirm that ILG carried out at positive gate voltage transforms *P*-SCO to *V*-SCO. The φ-scan of the *H*-SCO film reveals 4-fold symmetric *BM*-SCO (114) reflections which are offset from the STO (111) peaks by 45° (SI Figure S3b). This relative orientation between film and substrate leads to the smallest lattice mismatch that will be described later. On the other hand, *P*-SCO grows by a simple cube-on-cube relation on STO (001) as shown SI Figure S3c. For the *V*-SCO film, we hereafter reindex the *BM*-SCO (110) and (220) peaks as *V*-SCO (001) and (002) peaks, respectively. Cross-sectional scanning transmission electron microscopy (STEM)-high angle annular dark field (HAADF) measurements are conducted to confirm the structure and OVC orientations of the three films (SI Figure S4). *H*-SCO films show horizontal OVCs in which the Co ions within the OVCs are all located in tetrahedral sites. The well-known dimerization^14^ of the Co ions within the OVCs are clearly seen in the STEM images taken along the [100] zone axis (SI Figure S4a). This means that within the oxygen vacancy planes there are two distinct directions which are oriented at an angle of 90 deg relative to each other with different oxygen ion vacancy diffusivities.^38^ The *V*-SCO film reveals vertically oriented OVCs but the Co–Co dimerization cannot be seen along this zone axis. The *P*-SCO shows a conventional perovskite structure without any OVCs.

**Figure 1:**
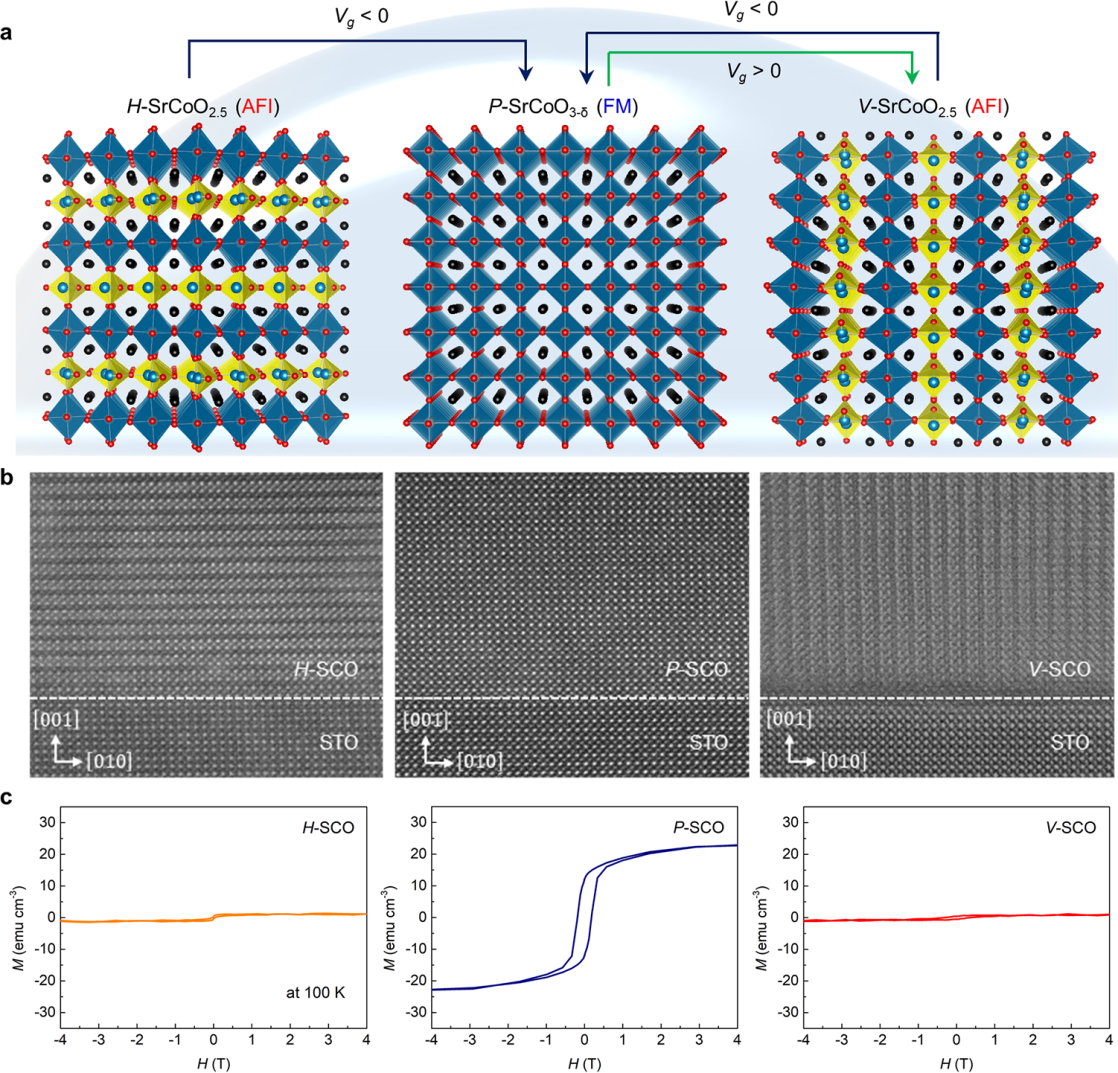
Electrical control of oxygen vacancy ordering in SrCoO_*x*_ thin films. (a) Crystal structure of brownmillerite SrCoO_2.5_ having horizontal OVCs (*H*-SCO) (left), perovskite SrCoO_3-δ_ (*P*-SCO) (center), and brownmillerite SrCoO_2.5_ having vertical OVCs (*V*-SCO) (right). The structure can be sequentially transformed by ionic liquid gating (ILG) at different gate voltages. *H*-SCO transforms to *P*-SCO at negative gate voltages (*V*_*g*_). Then, *P*-SCO transforms to *V*-SCO at positive *V*_*g*_. *V*-SCO is transformed back to *P*-SCO at negative *V*_*g*_. The black, blue, and red spheres denote Sr, Co, and O ions, respectively. The blue and yellow polyhedra represent octahedra and tetrahedra, respectively. (b) STEM-HAADF images of *H*-SCO, *P*-SCO, and *V*-SCO (from left to right) grown on STO (001) substrates. (c) Magnetization versus field curves for *H*-SCO, *P*-SCO, and *V*-SCO (from left to right) measured at 100 K.

Figure 1 summarizes the phase transformations of SCO_*x*_ induced by ILG. *H-*SCO transforms to *P-*SCO at negative gate voltage. Furthermore, positive gate voltage transforms *P-*SCO to *V-*SCO, which can then be reversibly transformed back to *P-*SCO by negative gate voltage (SI Figure S5). Thus, the orientation of the OVCs can be controlled by an electric field induced at the ILG/oxide interface, which also goes in parallel with significant changes of the physical properties of the film. As shown in Figure 1c, the magnetization versus in-plane field loop (*M-H*) collected at 100 K for *P*-SCO indicates ferromagnetism (FM), while the loops for *H*-SCO and *V*-SCO are consistent with the known antiferromagnetic (AFM) ground state of *BM*-SCO.^36^ Note that *P*-SCO transforms only to *V*-SCO but not to *H*-SCO no matter what the magnitude of the gate voltage is applied (SI Figure S2).

**Figure 2:**
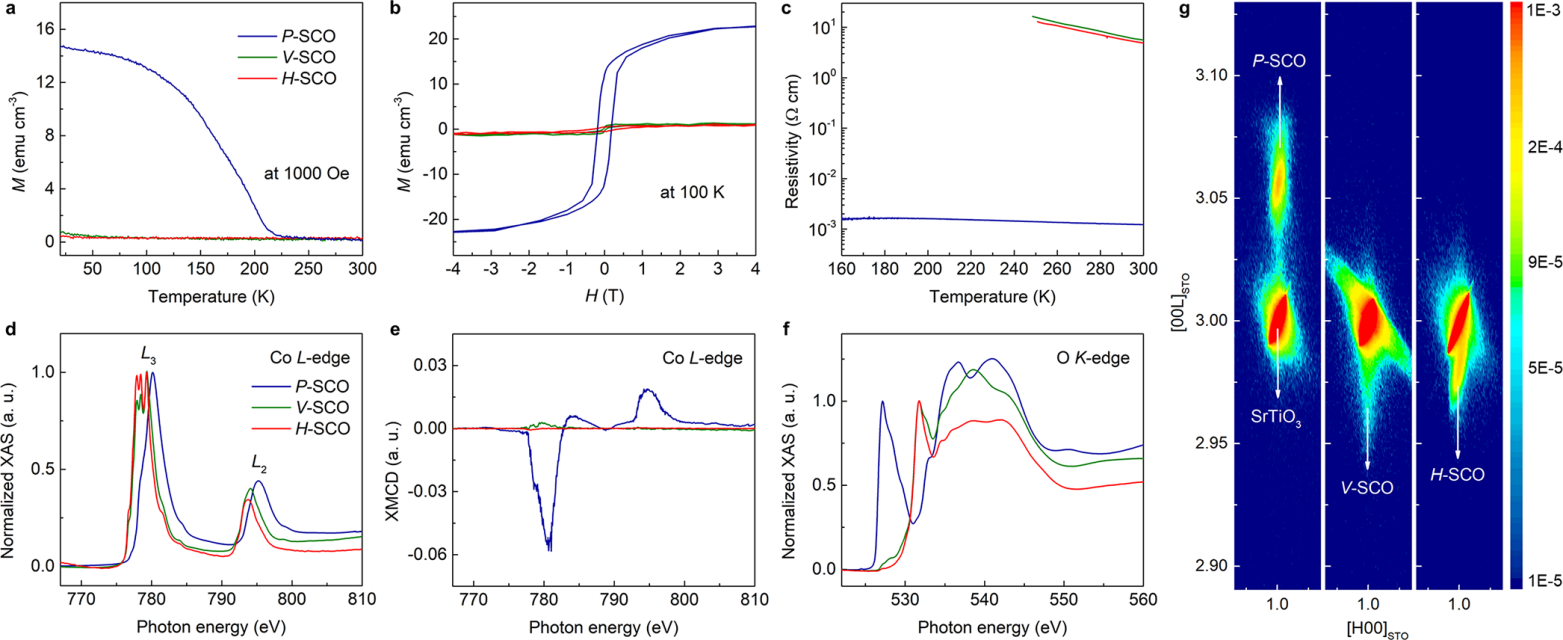
Electronic, magnetic, and structural properties of three distinct SrCoO_*x*_ films. (a) Temperature-dependent magnetization at 1000 Oe. (b) Magnetization versus in-plane magnetic field at 100 K. (c) Temperature dependent resistivity. (d) Normalized XAS spectra in the vicinity of the Co *L*_2,3_ absorption edge. (e) XMCD spectra recorded in remanence after applying an in-plane magnetic field of 6 T. (f) Normalized XAS O *K* edge spectra. XAS and XMCD were recorded in grazing incidence. (g) Reciprocal space maps of three SCO_*x*_ films on STO (001).

The three SrCoO_*x*_ phases discussed above have distinct physical properties. Temperature dependent magnetization versus temperature (*M-T*) curves recorded at 1000 Oe (Figure 2a) and magnetization versus external field (*M-H)* curves recorded at 100 K, mentioned above, show clear evidence for ferromagnetism only in *P*-SCO. The saturation magnetization (23 emu cm^–3^ or 0.15 μ_B_/Co) determined for the *P*-SCO film is smaller than that of bulk *P*-SCO.^37^ It is challenging to make a fully oxidized SrCoO_3_ thin film because the valence states of Co^2+^ and Co^3+^ are more stable than Co^4+^.^14^ Thus, the smaller magnetization is likely due to the incompletely oxidized state of the film. that is, SrCoO_3-δ_.^14^ Temperature-dependent resistivity curves (Figure 2c) show a substantial difference in resistivity at 300 K between *H*- and *V*-SCO, on the one hand, and *P*-SCO, on the other hand: the former being insulating, the latter metallic. X-ray photoelectron spectroscopy (XPS) measurements were carried out to distinguish the chemical states of Co and O in the three phases (SI Figure S6). XPS spectra reveal that the Co 2*p* core levels of *P*-SCO have a higher binding energy than those of *H*- and *V*-SCO, which is attributed to the higher degree of covalent character of the Co–O bond in *P*-SCO.^39,40^ O 1*s* XPS spectra show a distinct peak at the lower binding energy side in the case of *H*-SCO and *V*-SCO which originate from the oxygen deficient CoO_4_ tetrahedral layers, which is therefore missing in the case of *P*-SCO. Figure 2d compares X-ray absorption spectra (XAS) recorded in the vicinity of the Co *L*_2,3_ edge for the three phases at 2 K. The absorption spectra of *H*- and *V*-SCO are characterized by a similar multiplet structure at the *L*_3_ edge, while a small intensity difference between them might be due to the different OVC orientations between *H*- and *V*-SCO relative to the incident X-ray beam. Moreover, the peak shift (observed for both *L*_3_ and *L*_2_ peaks) toward higher energy in the *P-*SCO spectrum indicates an increased valence state of Co^14^ as compared to *H*- and *V*-SCO.

To complement these data, X-ray magnetic circular dichroism (XMCD) measurements were carried out at 2 K. Figure 2e shows the XMCD spectra collected in the total electron yield mode in the vicinity of the Co *L*_2,3_ edge in zero field after applying an in-plane magnetic field of 6 T. Only for *P*-SCO, a finite signal is observed related to its FM order, while for the AFM phases *H*- and *V*-SCO, no XMCD signal is observed within the experimental accuracy. Normalized O *K*-edge XAS spectra (Figure 2f) exhibit a distinct pre-edge peak at ∼527 eV for the *P*-SCO film, which originates from Co 3*d* – O 2*p* hybridization.^14^ On the other hand, *H*-SCO and *V*-SCO reveal very weak pre-edge peaks owing to the oxygen-deficiency and resulting suppression of such a hybridization. The XMCD hysteresis measurements (SI Figure S7) further confirm that a ferromagnetic hysteresis loop exists only for *P*-SCO. In summary, these studies reveal that the electronic, magnetic, and chemical properties between *H*- and *V*-SCO are similar, while they are distinctly different in the case of *P*-SCO.

The mutual crystallographic relation between the STO substrate and the film lattice was examined using X-ray diffraction by recording reciprocal space maps (RSMs) (Figure 2g) of the three phases. The highest intensity is observed for the bulk (103) reflection of the STO substrate. For all phases the in-plane component (H) of the reflection is aligned to that of the substrate indicating coherent strain without relaxation even after ILG. On the other hand, the out-of-plane component (L) of the reflection reveals a vertical relaxation in the case of *P*-SCO (peak positon at *L* = 3.06 reciprocal lattice units (rlu) versus 3.00 for STO). In the case of *V*-SCO there is almost no relaxation (the peaks nearly coincide) while for *H*-SCO the c-lattice parameter is almost four times larger than that of STO so that this reflection corresponds to L = 12.

A more in-depth analysis of the *H*- and *V*-SCO structure was carried out by using a GaJet X-ray source (λ = 1.3414 Å) and a six-circle X-ray diffractometer designed for the study of thin films and surfaces. Structure refinements were carried out using 33 and 22 symmetry independent reflections collected for *H*- and *V*-SCO, respectively. The detailed analysis is described in the SI. The *H*-SCO film can be viewed as an in-plane  superstructure with respect to the STO (001) substrate, while the *V*-SCO film corresponds to a (2 × 1) superstructure We find that the structure of *H*- and *V*-SCO can be mapped to the space groups *Ima2* and *Pmmm*, respectively. The lattice constants of *H*- and *V*-SCO films grown on STO (001) substrates are given in SI Table S1. SI Figure S8 shows a schematic view of the *H*-SCO structure, which is identical to the reported brownmillerite phase: the atomic coordinates agree to within 0.02–0.05 lattice units. For *V*-SCO, which is shown in SI Figure S9, we find an oxygen vacancy located at the sites labeled (1) and (2). These sites are approximately occupied by 50% each, corresponding to two (coherent) domains in which either site (1) or site (2) has a vacancy. This is due to the fact that beginning with the parent *P*-SCO (1 × 1) phase there are two symmetry equivalent sites to create a vacancy both leading to the (2 × 1) superstructure. In addition there are two rotational domains which are related to each other by a 90° rotation, resulting in a (2 × 1) and a (1 × 2) superstructure domain which, however, do not interfere in reciprocal space. In addition, there is a small (∼0.01 lattice units) relaxation of the Sr ions along the *a*-axis.

**Figure 3:**
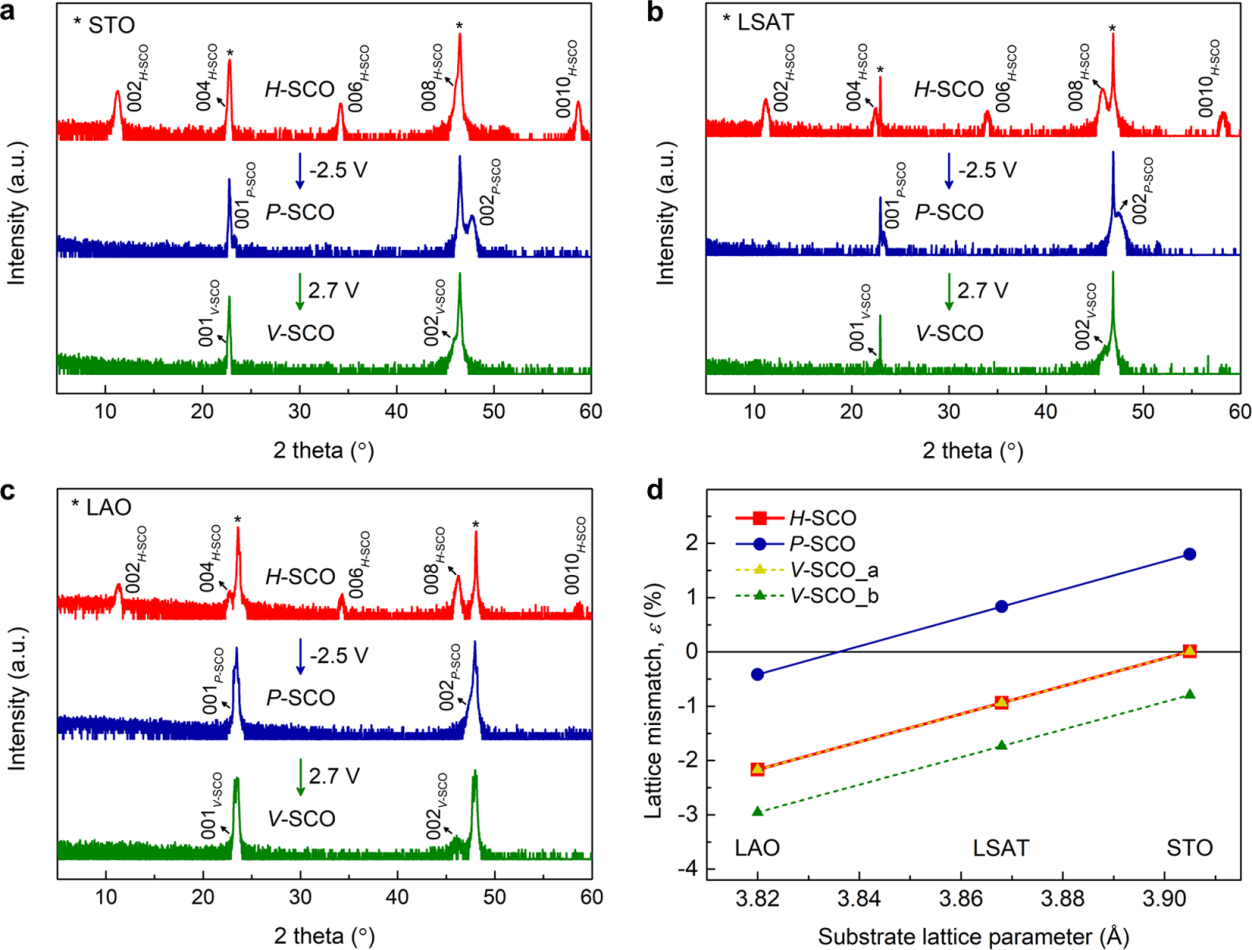
Substrate-independent oxygen vacancy ordering in SrCoO_*x*_ thin films. θ–2θ XRD scans for ionic liquid gated SCO_*x*_ films grown on (a) STO (001), (b) LSAT (001), and (c) LAO (001). Asterisks denote substrate peaks. All films grown on the three different substrates show the same phase transformation sequence from *H*-SCO to *P*-SCO at *V*_g_ = −2.5, followed by *P*-SCO to *V*-SCO at *V*_g_ = 2.7. (d) Lattice mismatch (ε) of the three phases for each substrate. The lattice mismatch was calculated based on the effective lattice constants of each phase, as summarized in SI Table S2. Positive (negative) lattice mismatch corresponds to tensile (compressive) strain in the film.

The ILG controlled OVC ordering was further investigated by employing three different, compressively strained films grown on an STO (001), LSAT (001), and LAO (001) substrate (see Figure 3). Since it is known that substrate-induced tensile strain leads to the growth of *V*-SCO,^41^ we have selected compressively strained films to investigate the ILG induced OVC ordering beginning with as deposited *H*-SCO films. All *H*-SCO films are found to follow the same ILG induced OVC ordering sequence, namely from *H*-SCO, to *P*-SCO, to *V*-SCO regardless of strain (Figure 3a-3c). Pseudocubic lattice constants based on the epitaxial relationship and the corresponding lattice mismatch between each phase and substrate are summarized in SI Table S2 and in Figure 3d, respectively. The in-plane lattice vectors of *H*-SCO film are rotated by 45° rotation relative to those of STO, while *V*-SCO shows anisotropic effective in-plane mismatches of ε_a_ and ε_b_ corresponding to the *V*-SCO [100] and [010] directions, respectively. Even though the lattice mismatch for *V*-SCO is larger than that of *H*-SCO for all substrates, oxygen removal from *P*-SCO by ILG always leads to the formation of *V*-SCO and not to *H*-SCO. Moreover, in the case of SCO films grown on LAO, the transformation from *P*-SCO to *V*-SCO exhibits a significant increase in strain. That is, the lattice mismatch increases from −0.4% (*P*-SCO/LAO) to −3% (ε_a_ of *V*-SCO/LAO). Thus, the OVC ordering induced by ILG occurs at the expense of the large strain. The time-dependent changes of source-drain current (*I*_SD_) during ILG and the subsequent phase change of SCO_*x*_ films on LSAT (001) are further described in SI Figure S10.

**Figure 4:**
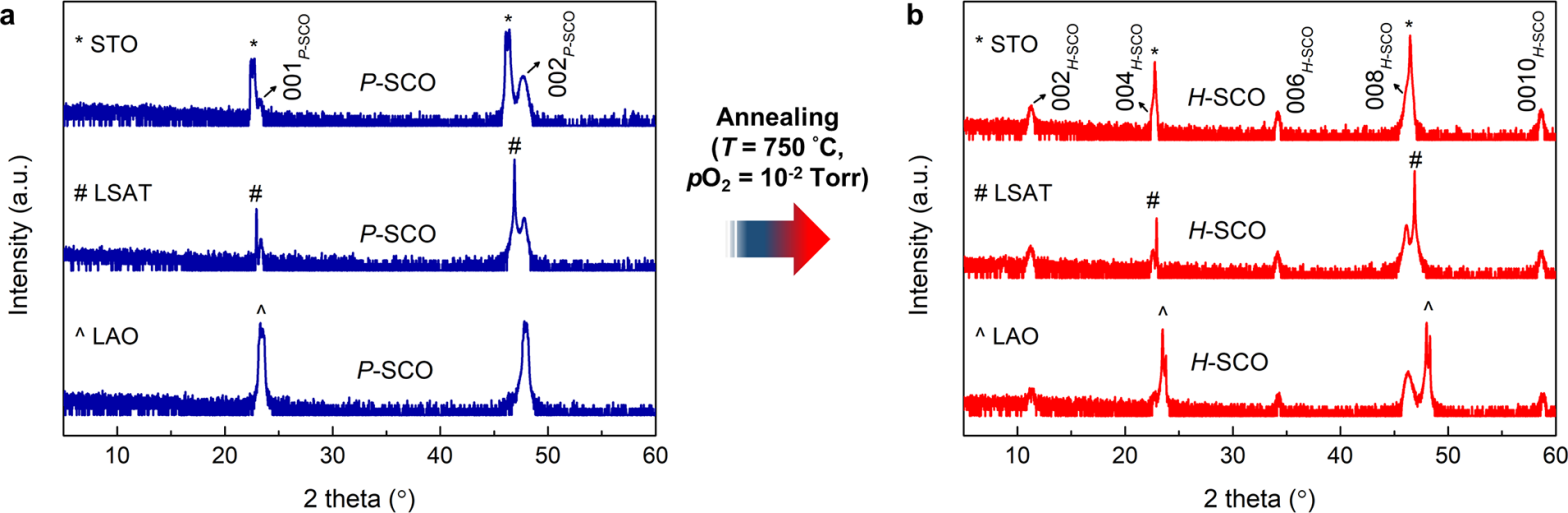
Phase transformations induced by thermal annealing. (a) θ–2θ XRD scans for 30 nm thick *P*-SCO films grown on three different substrates STO (001), LSAT (001), LAO (001). (b) θ–2θ XRD scans after annealing at 750 °C in an oxygen pressure (*p*O_2_) of 10^–2^ Torr for 30 min. All films are transformed from *P*-SCO to *H*-SCO after this annealing process.

**Figure 5:**
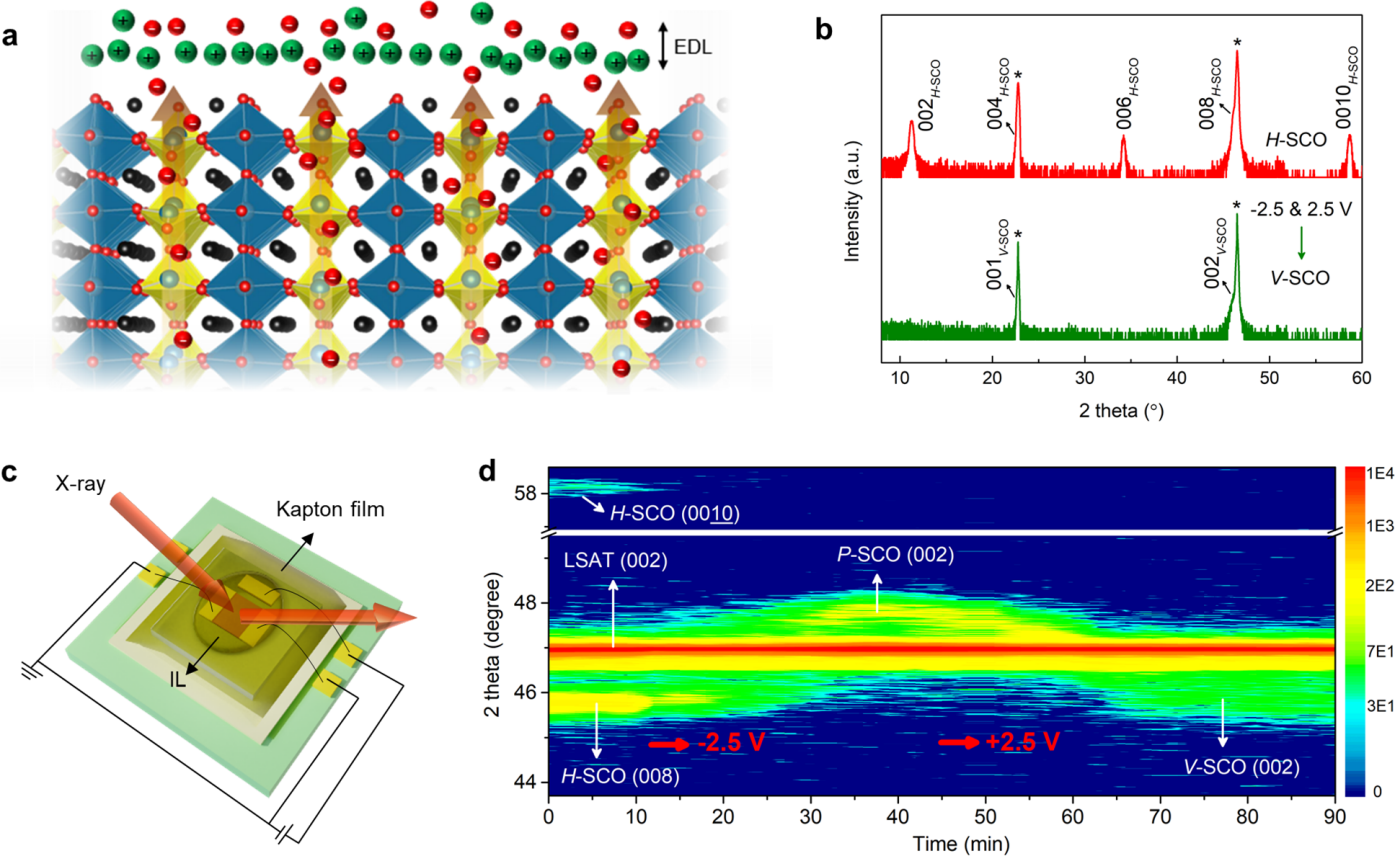
In situ monitoring of the electric-field control of oxygen vacancy ordering (a) Schematic representation of the formation of *V*-SCO in a *P*-SCO film by ILG at a positive gate voltage. The green and red spheres in EDL represent positive and negative ions, respectively. The black, blue, and red spheres in the lattice denote Sr, Co, and O ions, respectively. The blue and yellow polyhedra represent octahedra and tetrahedra, respectively. (b) θ–2θ scans of an as-deposited *H*-SCO/STO(001) film and the same film after it was subjected to negative voltage gating (−2.5 V) followed by positive voltage gating (2.5 V). This process leads to the formation of *V*-SCO via an intermediate *P*-SCO phase. Asterisks denote substrate peaks. (c) Schematic of an *in situ* XRD measurement during ILG. To thin down the IL on the film surface, a Kapton film was attached to the device after applying the IL. (d) Time dependent *in situ* XRD measurements of several reflections characteristic for the different phases during ILG of *H*-SCO/LSAT (001), demonstrating electrical control of the formation of OVCs. Red arrows indicate the onset of the applied gate voltage.

The phase transformation of *P*-SCO is further studied by an annealing treatment. *P*-SCO films grown on the three substrates were annealed at 750 °C in an oxygen pressure (*p*O_2_) of 10^–2^ Torr for 30 min. Figure 4 shows that all *P*-SCO films are transformed to *H*-SCO which is at variance with the ILG induced phase transformation. The phase change via ILG can be accounted for by the formation of an electric double layer (EDL) at the ionic liquid/oxide interface and the subsequent electric field induced migration of oxygen perpendicular to the surface.^42^ Thus, as depicted in Figure 5a, we judge that ILG of the *P*-SCO films leads to the migration of oxygen ions along a vertical pathway, resulting in vertically oriented OVCs. In contrast to our results, some other *BM* systems such as La_0.5_Sr_0.5_CoO_3-δ_^25^ and SrFe_0.5_Co_0.5_O_3-δ_^32^ have shown horizontal OVC ordering from the gating of the perovskite phase. The substitution of Co by Fe and Sr by La significantly changes the film properties such as chemical stability, magnetism, and OVC ordering.^25−27^ For example, SrFeO_2.5_ films grown on STO (001) were found to develop vertical or horizontal OVC ordering just by changing the deposition conditions.^27^ Thus, depending on the chemical composition of the *BM* phase and the gating conditions, distinctly different behavior of the OVC ordering is expected. Their investigation is subject to further studies.

Films that have a *H-*SCO structure can be transformed into the *V*-SCO phase by a sequence of two ILG steps (Figure 5b). Finally, we demonstrate *in situ* control of the OVCs by employing a specially designed *in situ* XRD setup using a device that was fabricated as illustrated in SI Figure S11 and that is depicted in Figure 5c. As shown in Figure 5d, the pristine *H*-SCO film shows the (0010) and the (008) reflection at 2θ∼58° and 45.8°, respectively. Upon gating with a positive gate voltage during the *H*-SCO to *P*-SCO transformation these peaks gradually disappear within about 20–25 min while the (002) peak of the *P*-type film appears after about 25 min with almost no time overlap. Subsequently, negative gate voltage biasing leads to the formation of the *V*-SCO phase as concluded by the appearance of the *V*-SCO (002) peak and the simultaneous disappearance of the *P*-SCO (002) reflection. Also, there is no trace of the *H*-SCO (0010) reflection.

## Conclusion

In summary, we have demonstrated a method for controlling the orientation of the oxygen vacancy channels in thin oxide films, here of the exemplary transition metal oxide SrCoO_*x*_ irrespective of strain in the film that would otherwise favor horizontally ordered OVCs. This concept can be used for a wide range of diverse device applications.

## Methods

### Sample Preparation

Thirty nm thick epitaxial SCO films were grown on several substrates by pulsed laser deposition (Pascal Co., Ltd.) using a 248 nm KrF excimer laser source. LAO, LSAT, and STO (001) substrates with a size of 10 × 10 mm^2^ were used. *H-*SCO films were grown at 770 °C in an oxygen pressure of 10 mT. *P*-SCO films were grown at 770 °C using an oxygen pressure (*p*O_2_) of 300 mT, and then postannealed at 770 °C in 500 Torr (*p*O_2_) for 30 min. To carry out ILG of the films, the samples were cut into small chiplets, each with an area of ∼3.3 × 5 mm^2^, using a diamond scriber.

ILG was carried out in a polytetrafluoroethylene (PTFE) boat in which the gate electrode was formed from a 0.5 mm thick Au foil (5 × 4 mm^2^) that was placed adjacent to the chiplet (SI Figure S1). Both the chiplet and Au foil were covered with an ionic liquid, DEME-TFSI (*N*,*N*-diethyl-*N*-methyl-*N*-(2-methoxyethyl) ammonium bis(trifluoromethanesulfonyl)imide). After gating, the liquid was removed by placing the sample in a beaker of acetone followed by isopropyl alcohol. For the kinetics measurements, source-drain current (*I*_SD_) was measured during gating of SCO/LSAT (SI Figure S10).

### Device Fabrication

Standard photolithographic methods were used to fabricate devices for transport measurements. Channels, 65 × 30 μm^2^ in area, were defined by a positive tone ARP3540 resist. The SCO_*x*_ films were subsequently etched using a mixture of 10 mL water and 1 mL phosphoric acid (85%). Then, electrical contact pads were formed from Au (70 nm)/Ru (5 nm) bilayers by a lift-off technique. The Ru and Au were deposited by ion beam sputtering (SCIA coat 200).

*In situ* XRD measurements were carried out using a portion of an *H*-SCO film grown on LSAT (001). The chiplet used had an area of 5 × 5 mm^2^. A channel with an area of 2 × 2 mm^2^ was defined and then Au (70 nm)/Ru (5 nm) electrodes were formed. A gate electrode with an area of 0.8 × 2 mm^2^ was formed on the chiplet next to the device itself (see SI Figure S11). The device was attached to the sample holder using double-side tape. After placing the ionic liquid on the device surface, a Kapton film was attached to reduce its thickness.

### Characterization

Temperature and magnetic field dependent magnetization was measured using a MPMS3 SQUID (Superconducting Quantum Interference Device) magnetometer. Transport measurements were carried out in a physical property measurement system (Quantum Design). The gate voltage was applied using a Keithley 2450A source meter. The film resistance was measured using a constant current of 500 nA (Keithley 6221 current source), and the voltage was measured using a Keithley 2182A nanovoltmeter. θ–2θ scans, *in situ* XRD, and RSM were performed using a Bruker D8 Discover X-ray diffractometer and Cu–Kα radiation. A GaJet X-ray source (λ = 1.3414 Å) XRD was used to analyze the detailed structure of the *H*- and *V*-SCO thin films as described in the SI. STEM-HADDF measurements were carried out in a FEI Titan 80–300 transmission electron microscope with an electron beam energy of 300 kV. X-ray absorption spectroscopy (XAS) and X-ray magnetic circular dichroism (XMCD) measurements were conducted at the vector cryomagnet XMCD end station of the BOREAS beamline at the ALBA synchrotron source.^43^ The measurements were carried out at a base pressure of <1 × 10^–10^ mbar and under an applied magnetic field up to 6 T along the incident beam direction. Co *L*_2,3_ and O *K* edge spectra were collected at grazing incidence (70° off normal) at 2 K, using total electron yield (TEY) detection. Nearly 100% circularly polarized left and right X-rays were formed using an elliptically polarizing undulator. Additionally, element-specific hysteresis loops were performed by recording the TEY signal at the maximum value of the XMCD at the *L*_3_ edge and at a pre-edge energy for normalization purposes while sweeping the magnetic field, for both X-ray polarizations. X-ray photoelectron spectroscopy (XPS) measurements were made using a K-Alpha Thermo Scientific instrument. The film surface was gently cleaned by an Ar cluster ion etching prior to the measurement.

## Supporting Information Available

Structure refinements of *H*-SCO and *V*-SCO. Schematic diagrams of the ILG experiment. Gate voltage dependent resistivity and structural changes, Phi scans, High magnification STEM-HAADF images, XMCD hysteresis loop, XPS, lattice parameter information, Time-dependent changes of *I*_SD_ during gating, Schematic of device fabrication for *in situ* XRD measurements (PDF)
