# Supplementary material for: Control of Oxygen Vacancy Ordering in Brownmillerite Thin Films via Ionic Liquid Gating
Source: ACS Nano. 2022 Apr 4;16(4):6206–14. doi: 10.1021/acsnano.2c00012 (PMC9047007; doi:10.1021/acsnano.2c00012)
Supplement: Supplementary file 1 — nn2c00012_si_001.pdf [file nn2c00012_si_001.pdf]

Supporting Information for

# Control of oxygen vacancy ordering in brownmillerite thin films *via* ionic liquid gating

Hyeon Han[1,†], Arpit Sharma[1,†], Holger L. Meyerheim[1], Jiho Yoon[1], Hakan Deniz[1], Kun-Rok Jeon[1], Ankit K. Sharma[1], Katayoon Mohseni[1], Charles Guillemard[2], Manuel Valvidares[2], Pierluigi Gargiani[2], Stuart S. P. Parkin[1*]

[1]Max Planck Institute of Microstructure Physics, 06120 Halle (Saale), Germany.
[2]ALBA Synchrotron Light Source, E-08290 Cerdanyola del Vallès, Barcelona, Spain.
[†]These authors contributed equally to this work.
*e-mail: stuart.parkin@mpi-halle.mpg.de

**Table of Contents:**

Supporting Text
Figure S1 to S9
Table S1

## Supporting text

### Structure refinements of H-SCO and V-SCO

Structural analyses of the *V*-$SrCoO_{2.5}$ (*V*-SCO) and *H*-$SrCoO_{2.5}$ (*H*-SCO) phases were carried out using a laboratory GaJet X-Ray source ($\lambda$=1.3414 Å) and a six-circle x-ray diffractometer especially designed for probing (ultra-) thin epitaxial films and surfaces. The data collection was carried out under grazing incidence of the incoming beam (incidence angle $\mu$=1 deg.) and by recording integrated intensities in transverse scans, *i.e.* by rotating the sample about its surface normal.

For *H*-SCO, 33 reflections were collected in total, which reduce to 26 by symmetry equivalence according to the 2mm plane group symmetry. For *V*-SCO we collected 22 reflections reducing to 17 by symmetry using the same point group symmetry. The average agreement of symmetry equivalent reflections is in the range of several percent. Subsequently, the squared structure factor amplitudes ($|Fobs|^2$) were derived by applying instrumental correction factors using standard crystallographic techniques (*1*). The structure analysis was carried out by least squares refinement of the $|Fobs|^2$ to the calculated ones ($|Fcalc|^2$) using the programs Shelx (*2*) and Prometheus (*3*) by allowing the atomic positions (x,y,z), site occupancy ($\theta$) and atomic displacement parameters (ADP) to vary according to the space groups Pmmm (*V*-SCO) and Ima2 (*H*-SCO) (*4*). In addition, the refinement requires the consideration of the domain structure. There are two rotational structure domains for H-SCO that involve the overlap of reflections making an incoherent averaging of the reflection intensities reflected by each domain necessary. The refinement quality is measured by the unweighted residuum (Ru) and the Goodness of Fit parameter (*5*) which were found to be excellent in both cases. For *V*-SCO we derived Ru=0.03 (GOF=0.80), for *H*-SCO Ru=0.07 (GOF=1.13).

Figs. S8 and S9 show the schematic structural models for *H*-SCO and *V*-SCO. Here, blue, green and red balls represent the Co, Sr, and O ions, respectively. *H*-SCO can be considered as an approximate $\left(\sqrt{2}\times\sqrt{2}\times 4\right)$ superstructure with respect to the bulk STO (001) substrate, while *V*-SCO corresponds to a $\left(\sqrt{2}\times 1\times 1\right)$ superstructure (see also below). In Figure S8, we compare the film structure with the structural model published in Ref. [6]. This is achieved by superimposing the bulk model (balls in the foreground) on the structural model of the film (balls

in the background). It is clearly evident that there exist only minor differences, especially near the Co atoms at z=1/4, but these lie within the experimental uncertainty. Figure S9 shows the model of the *V*-SCO film structure, which is characterized by the presence of two vacancy sites labelled (1) and (2) which in the case of the bulk *P*-SCO are symmetrically equivalent. The refinement indicates that there are two structural domains which are characterized by the presence of one or other vacancy. In addition to the presence of the vacancy, there is a lateral relaxation of the Sr atoms along the a-axis.

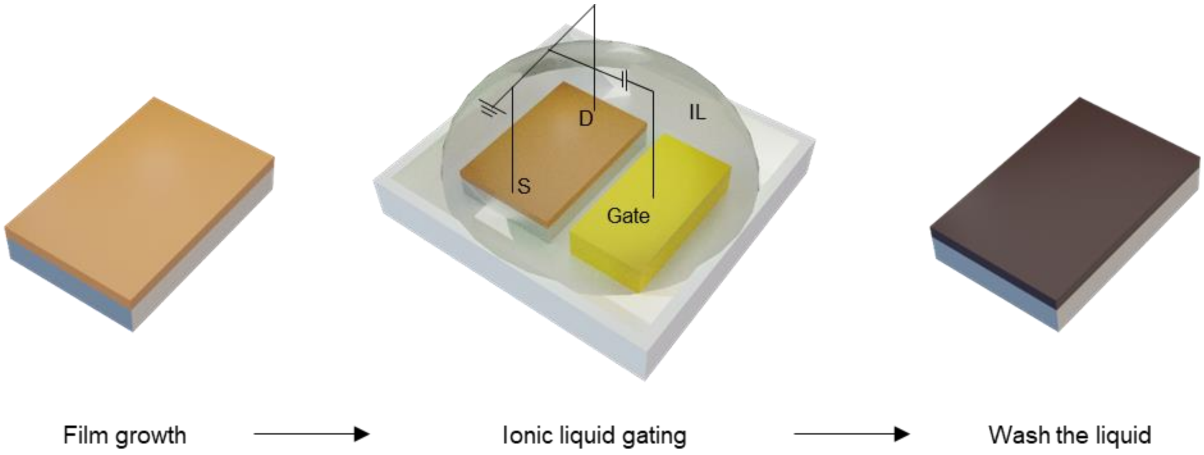


**Figure S1. Schematic diagram of ionic liquid gating of $SCO_x$ films.** The $SCO_x$ films were grown using pulsed laser deposition (PLD). The growth conditions are shown in Methods. The grown film was gated in a polytetrafluoroethylene (PTFE) boat using an adjacent gold plate (gate electrode) both covered by the ionic liquid DEME-TFSI (N,N-diethyl-N-methyl-N-(2-methoxyethyl) ammonium bis(trifluoromethanesulfonyl)imide). The film thickness is approximately equal to 30 nm and the lateral sample size is 3.33 x 5 $mm^2$. After gating, the IL was washed off by acetone and then isopropyl alcohol (IPA). For the kinetics measurements, source-drain current ($I_{SD}$) was measured during gating (Figure S10).

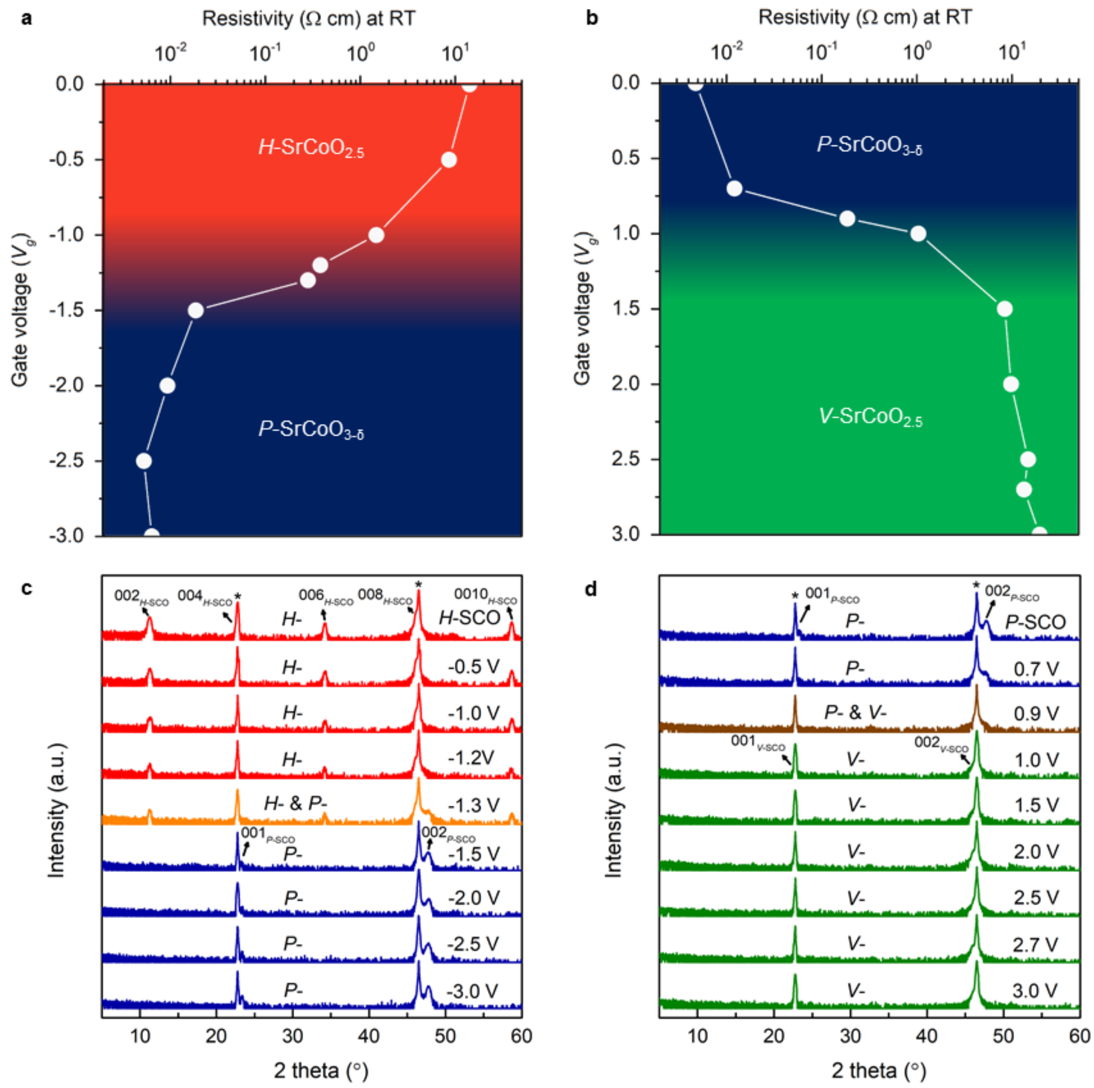


**Figure S2. Gate voltage dependent resistivity and structural changes of $SrCoO_x$ films.** Gate voltage dependent phase diagrams and resistivity changes of (a) *H*-SCO and (b) *P*-SCO thin films, and the corresponding $\theta-2\theta$ XRD scans for (c) *H*-SCO and (d) *P*-SCO, respectively. Negative voltages were applied to insulating *H*-SCO films to insert oxygen into the film, resulting in the transition to metallic *P*-SCO. Positive voltages were applied to metallic *P*-SCO films to extract oxygen from the film, leading to insulating *V*-SCO. Ionic liquid gating was performed during a period of 1.5 h for each voltage. Resistivity measurements of the gated films were performed by the van der Pauw method at room temperature. Asterisks denote substrate peaks.

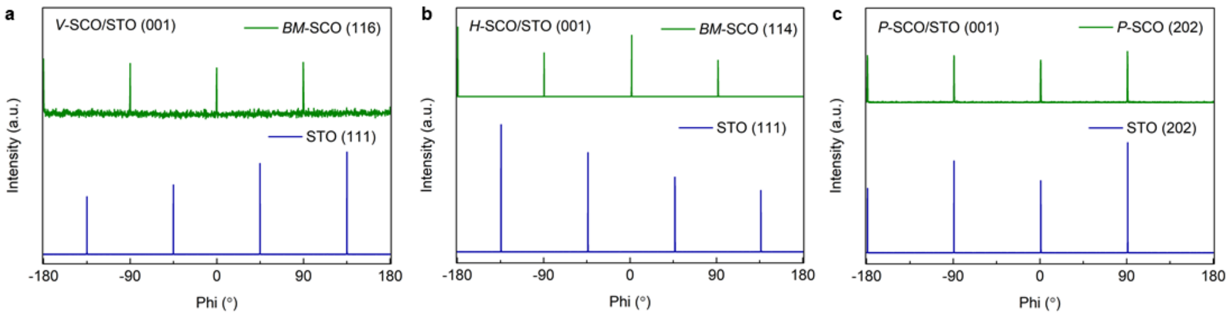


**Figure S3. Non-specular phi** (*φ*) **scans for** $\mathbf{SrCoO_x}$ **films.** Phi (*φ*) scans of the (a) *V*-SCO, (b) *H*-SCO, and (c) *P*-SCO thin films on STO (001). The angle chi (χ) in the four-circle geometry of the diffractometer was set to approximately 56, ~42, and ~45 degrees in (a), (b), and (c), respectively. There is a well-defined epitaxial relationship between the film and substrate lattice characterized by a 45 degrees shift between the pyramidal crystal faces {11L} and the STO octahedral {111} faces. For the case of the *H*-SCO film this results in a small lattice mismatch. The *P*-SCO film shows cube-on-cube growth on the substrate.

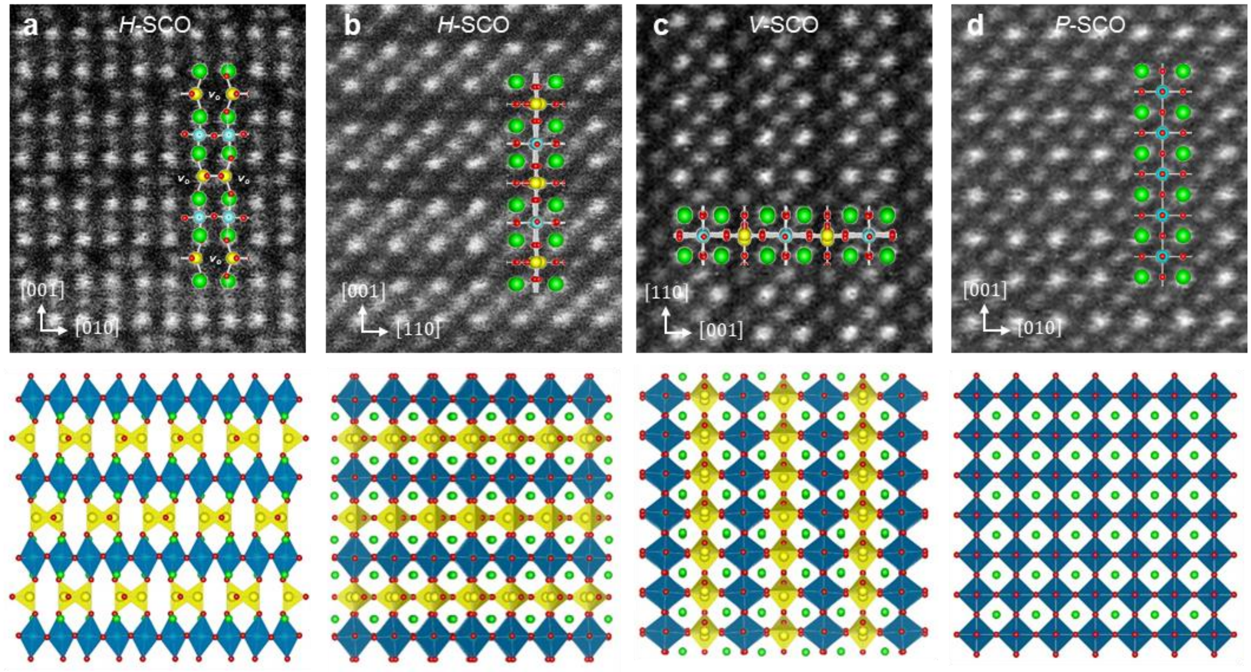


**Figure S4. High magnification STEM-HAADF images of three $SrCoO_x$ films.** STEM-HAADF images and the crystal structure of (a) *H*-SCO film projected along *BM*-SCO [100], (b) *H*-SCO film projected along *BM*-SCO $[1\bar{1}0]$ , (c) *V*-SCO film projected along *BM*-SCO $[\bar{1}10]$, and (d) *P*-SCO film projected along *P*-SCO [100]. The green, yellow, sky blue, and red spheres denote Sr ions, Co ions at the tetrahedral site, Co ions at the octahedral site, and O ions, respectively. The blue and yellow polyhedral represent octahedra and tetrahedra, respectively.

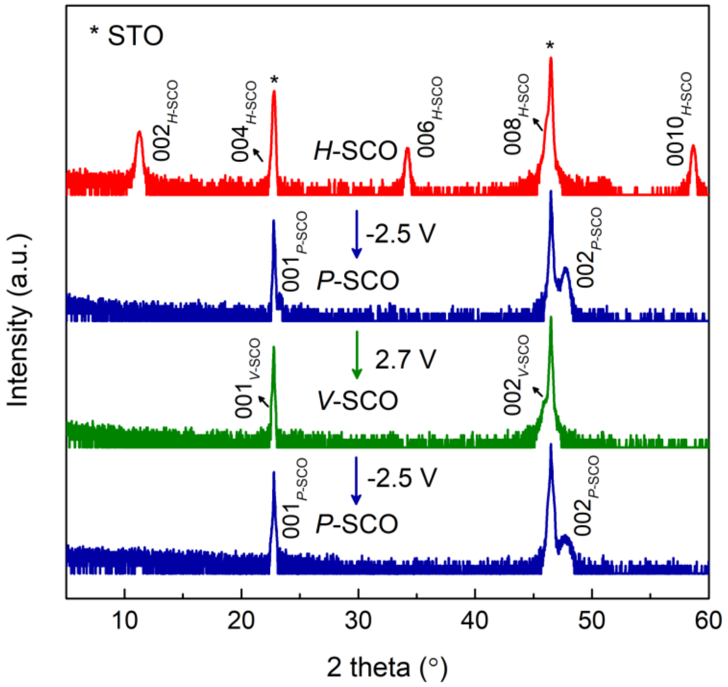


**Figure S5. Sequential OVC ordering and reversible control.** $\theta$–$2\theta$ XRD scans of $SCO_x$/STO at different gate voltages. *H*-SCO transforms to *P*-SCO by negative voltage gating, then *P*-SCO transforms to *V*-SCO by positive voltage gating. The *V*-SCO can be reversibly transformed back to *P*-SCO by negative voltage gating. Asterisks denote substrate peaks.

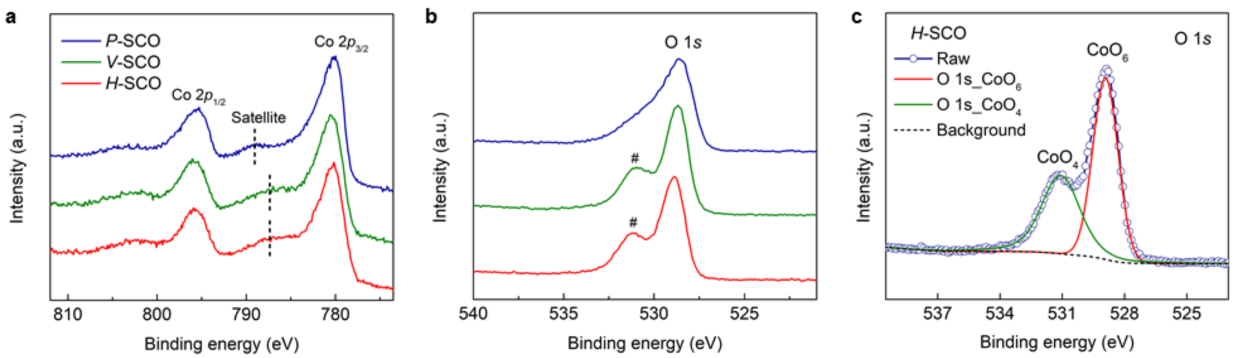


**Figure S6. XPS analysis of three $SrCoO_x$ films.** (a) Co 2*p* XPS spectra. *P*-SCO shows higher binding energy of the satellite peak (marked with vertical dashed lines in the figure) compared to *V*- and *H*-SCO, which is due to the greater covalent character of $SrCoO_{2.5}$ (Ref. 30, 31 in the main manuscript). (b) O1*s* XPS spectra. *V*-SCO and *H*-SCO show distinct higher binding energy peaks (# marks in the figure), which comes from oxygen deficient regions. *i.e.*, oxygen vacancy channels within the $CoO_4$ tetrahedral network. *H*-SCO and *V*-SCO shows nearly the same chemical states as expected. (c) Deconvolution of the O1*s* spectra. The portion of the higher binding energy region is ~42 %, which corresponds to the oxygen ion portion of $CoO_4$ tetrahedra in the $SrCoO_{2.5}$ lattice.

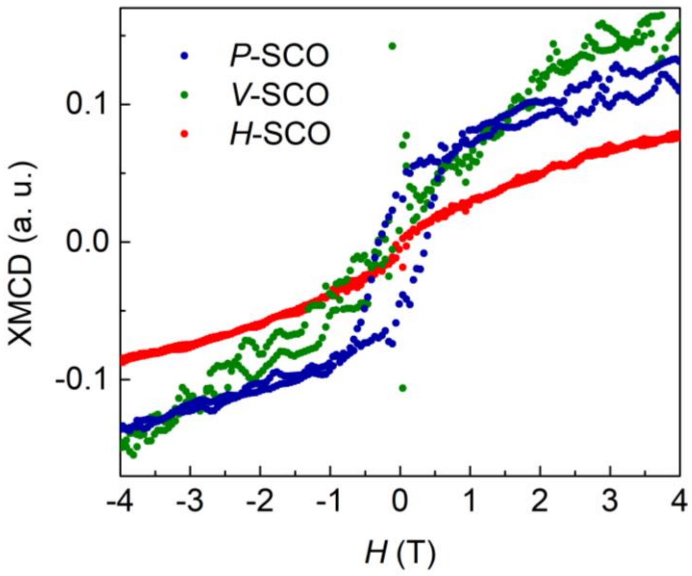


**Figure S7. XMCD Hysteresis loops of three $SrCoO_x$ films.** Hysteresis loops extracted from the maximum of the XMCD signal at the Co $L_3$ edge. Only *P*-SCO film shows evidence for a ferromagnetic hysteresis loop.

**Table S1. Lattice constants of $SrCoO_x$ thin films grown on $SrTiO_3$ (001) substrates.** The lattice constants of $SCO_x$ films as derived from the XRD measurements. The *V*-SCO axes *a*, *b* and *c* are parallel to that of the cubic STO substrate, while *a* and *b* of *H*-SCO are rotated by 45°. The space groups of *V*- and *H*-SCO structure are *Pmmm* and *Ima2*, respectively.

| | *a* (Å) | *b* (Å) | *c* (Å) |
|---|---|---|---|
| Bulk *BM*-$SrCoO_{2.5}$ | 5.470 | 5.574 | 15.745 |
| *H*-$SrCoO_{2.5}$ | 5.522 | 5.522 | 15.711 |
| *V*-$SrCoO_{2.5}$ | 7.810 | 3.905 | 3.947 |
| *P*-$SrCoO_3$ | 3.905 | 3.905 | 3.807 |

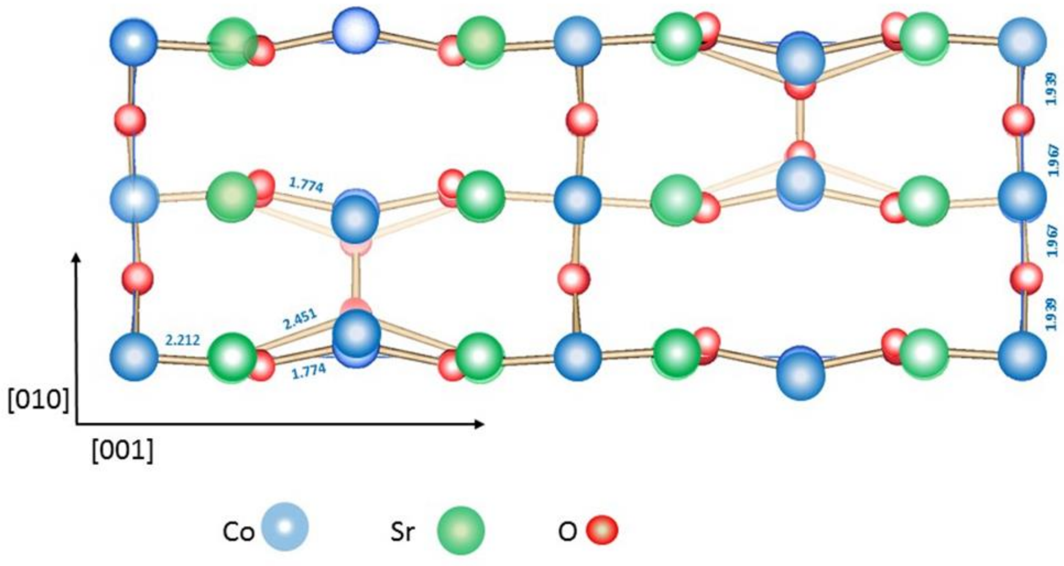


**Figure S8. Structure model of *H*-SCO in projection along the a-axis.** Blue, green, and red circles correspond to Co, Sr, and O ions, respectively. The image compares the refined film structure (balls in the background) with that of the bulk structure (balls in the foreground) according to the analysis of Ref. [6]. Only minor differences are observed. Numbers label interatomic distances in Ångström units.

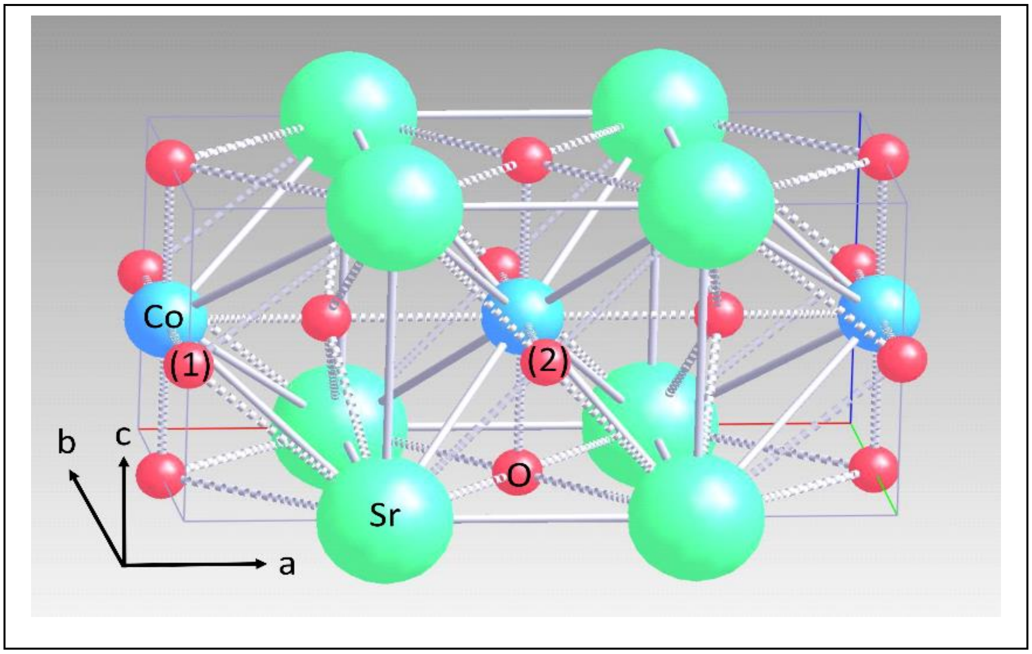


**Figure S9. Structure model for *V*-SCO.** Color code of the atoms is the same as in Figure S8. Note that the oxygen vacancies in sites labelled by (1) and (2) are about equally populated**.**

**Table S2. Pseudo-cubic lattice constants of bulk $SrCoO_x$ and substrates for lattice mismatch calculations.** For the lattice mismatch calculations, *H*-SCO, *V*-SCO, and *P*-SCO lattice constants are determined based on the bulk lattice parameters of *BM*-SCO (Ref. 27 in the main manuscript) and *P*-SCO (Ref. 28 in the main manuscript). The pseudo-cubic lattice constants of *H*-SCO were calculated taking into account in-plane a 45 degree rotation of *BM*-SCO relative to the substrate. The pseudo-cubic lattice constants of *V*-SCO were calculated considering the out-of-plane growth direction of *BM*-SCO [110], resulting in two different in-plane constants of $a_p$ and $b_p$ corresponding to *V*-SCO [100] and [010] directions, respectively. The substrate dependent lattice mismatches are presented in Figure 3d.

| | $a_p$ (Å) | $b_p$ (Å) | $c_p$ (Å) | Remark |
|---|---|---|---|---|
| *H*-$SrCoO_{2.5}$ | 3.905 | 3.905 | 3.936 | In-plane 45 degree rotation<br>$a_p = b_p = (a_{BM}^2+b_{BM}^2)^{0.5}$, $c_p = c_{BM,}/4$ |
| *V*-$SrCoO_{2.5}$ | 3.936 | 3.905 | 3.905 | Out-of-plane is *BM*-SCO [110]<br>$a_p = c_{BM}/4$, $b_p = c_p= (a_{BM}^2+b_{BM}^2)^{0.5}$ |
| *P*-$SrCoO_3$ | 3.836 | 3.836 | 3.836 | |
| $SrTiO_3$ | 3.903 | 3.903 | 3.903 | Substrate |
| LSAT | 3.868 | 3.868 | 3.868 | Substrate |
| $LaAlO_3$ | 3.820 | 3.820 | 3.820 | Substrate |

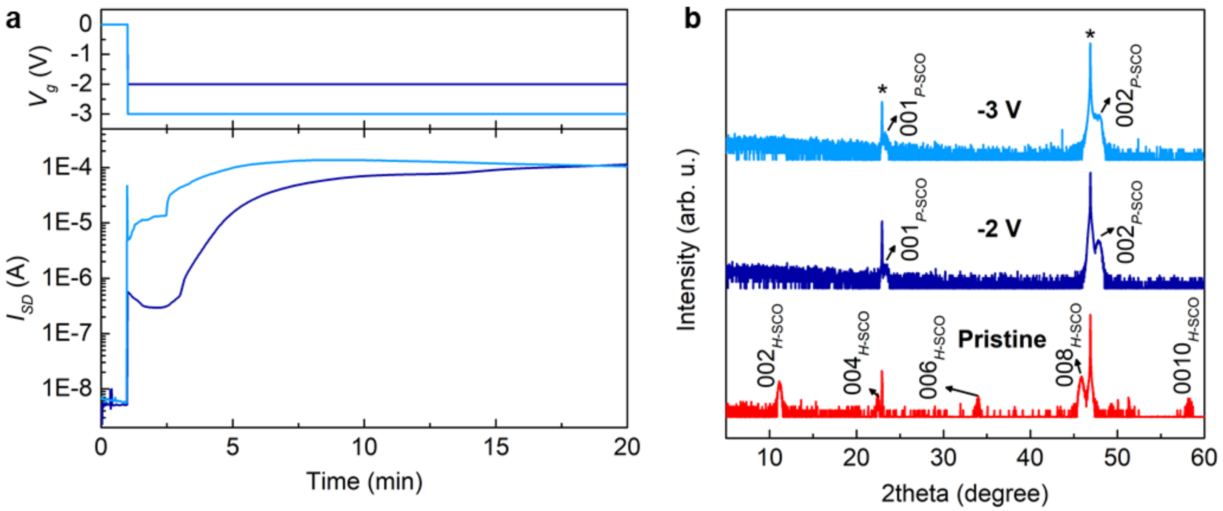


**Figure S10. Time-dependent changes of source-drain current ($I_{SD}$) during ILG and the subsequent phase transitions.** (a) Time-dependent changes of $I_{SD}$ during ILG with gate voltages of -2 V (dark blue) and -3 V (light blue), respectively. 3.3×5 $mm^2$ the *H*-SCO films grown on the LSAT (001) substrate was used. (b) $\theta$–$2\theta$ XRD scans of the pristine and the gated samples. Asterisks denote substrate peaks. The increase of $I_{SD}$ indicates metallization resulting from oxygen migration into the insulating *H*-SCO, forming the *P*-SCO phase which takes place on a time scale of about 10 minutes. A relaxation of the leakage current is seen at the start of gating. The XRD data further confirm the phase transformation via ILG. The phase changes via ILG is processed by electric-double layer (EDL) formation at the ionic liquid/oxide surface and the subsequent ion migration. Because the EDL formation takes only a few msec,[8] the gating kinetics mainly determined by the oxygen ion migration into the film lattice.

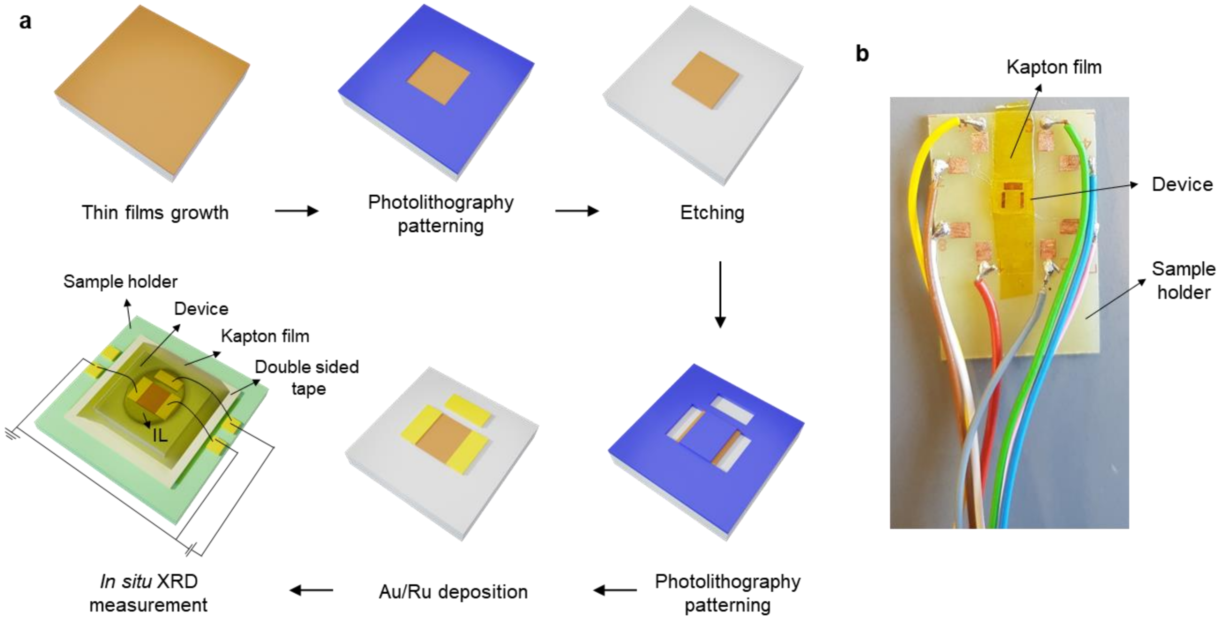


**Figure S11. Schematic of device fabrication for *in situ* XRD measurements.** (a) Standard photo-lithography was employed to fabricate the device. The chiplet used had an area of 5×5 mm$^2$. A channel with an area of 2×2 mm$^2$ was formed and then Ru (~5 nm)/ Au (~70 nm) electrodes were formed. The gate electrode with an area of 0.8×2 mm$^2$ was formed on the chiplet from the same Ru/Au bilayer and was placed adjacent to the device itself . The device was attached to the sample holder using double-sided tape. After placing the IL on the device surface, a Kapton film was attached to thin the IL. The contact wires were connected, then the ILG was performed during the XRD measurement. (b) A photo of the ionic liquid device for the *in situ* XRD measurement.

**References in Supporting Information**


1. M.M Woolfson "An Introduction to X-Ray Crystallography" (Cambridge University Press, Cambridge, 1979).
2. G.M. Sheldrick, Crystal structure refinement with SHELXL, *Acta. Cryst.*, **C71**, 3-8 (2015).
3. U. H. Zucker, E. Perenthaler, W. F. Kuhs, R. Bachmann and H. Schulz. Prometheus: A program system for investigation of anharmonic thermal vibrations in crystals, *J. Appl. Cryst.* **16**, 358 (1983).
4. International tables for X-ray crystallography. Vol. 1, Symmetry groups, N F M Henry; Kathleen Lonsdale, Dame; International Union of Crystallography. (Birmingham, 1969) Published for the International Union of Crystallography by the Kynoch Press.
5. Abrahams S, Indicators of Accuracy in Structure Factor Measurement. *Acta. Cryst.* **A25**, 165 (1969).
6. A. Muñoz, *et al.* Crystallographic and magnetic structure of $SrCoO_{2.5}$ brownmillerite: Neutron study coupled with band-structure calculations. *Physical Review B* **78**, 054404 (2008).
7. Y. Long, Y. Kaneko, S. Ishiwata, Y. Taguchi, Y. Tokura, Synthesis of cubic $SrCoO_3$ single crystal and its anisotropic magnetic and transport properties. *J Phys Condens Matter* **23**, 245601 (2011).
8. T. Jänsch, J. Wallauer, B. Roling, Influence of Electrode Roughness on Double Layer Formation in Ionic Liquids. *J. Phys. Chem. C* **119**, 4620-4626 (2015).
